# The Brain Renin-Angiotensin System and Mitochondrial Function: Influence on Blood Pressure and Baroreflex in Transgenic Rat Strains

**DOI:** 10.1155/2013/136028

**Published:** 2013-01-21

**Authors:** Manisha Nautiyal, Amy C. Arnold, Mark C. Chappell, Debra I. Diz

**Affiliations:** ^1^Hypertension and Vascular Research Center, Wake Forest University School of Medicine, Medical Center Boulevard, Winston-Salem, NC 27157-1032, USA; ^2^Department of General Surgery, Wake Forest University School of Medicine, Medical Center Boulevard, Winston-Salem, NC 27157-1032, USA

## Abstract

Mitochondrial dysfunction is implicated in many cardiovascular diseases, including hypertension, and may be associated with an overactive renin-angiotensin system (RAS). Angiotensin (Ang) II, a potent vasoconstrictor hormone of the RAS, also impairs baroreflex and mitochondrial function. Most deleterious cardiovascular actions of Ang II are thought to be mediated by NADPH-oxidase- (NOX-) derived reactive oxygen species (ROS) that may also stimulate mitochondrial oxidant release and alter redox-sensitive signaling pathways in the brain. Within the RAS, the actions of Ang II are counterbalanced by Ang-(1–7), a vasodilatory peptide known to mitigate against increased oxidant stress. A balance between Ang II and Ang-(1–7) within the brain dorsal medulla contributes to maintenance of normal blood pressure and proper functioning of the arterial baroreceptor reflex for control of heart rate. We propose that Ang-(1–7) may negatively regulate the redox signaling pathways activated by Ang II to maintain normal blood pressure, baroreflex, and mitochondrial function through attenuating ROS (NOX-generated and/or mitochondrial).

## 1. Introduction

**Figure 1:**
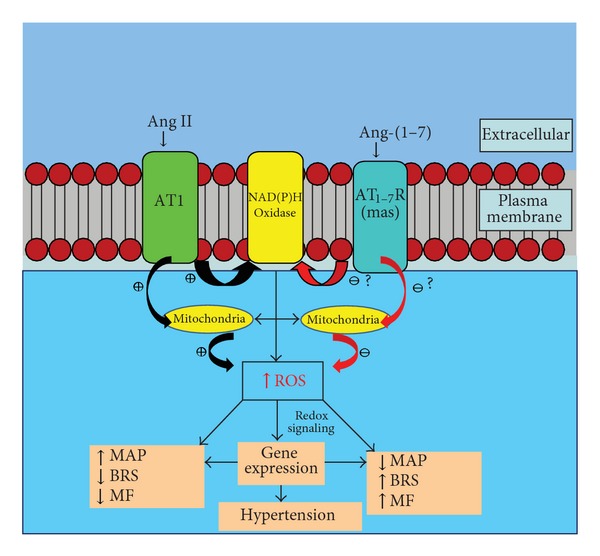
Proposed model: Ang-(1–7) through indirect (NADPH oxidase-mediated ROS) and/or direct interactions with mitochondria can attenuate ROS in dorsal medulla resulting in reduced blood pressure and enhanced baroreflex and mitochondrial function. ROS: reactive oxygen species; MAP: mean arterial pressure; BRS: baroreflex sensitivity; MF: Mitochondrial function; ?: not known.

**Figure 2:**
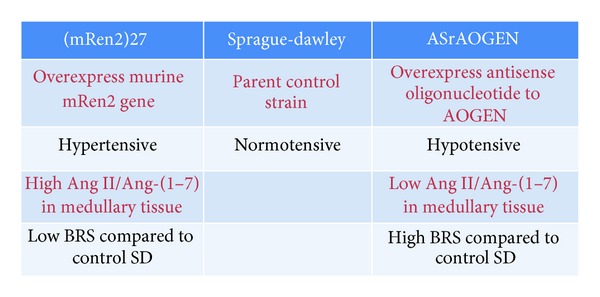
Major characteristics of (mRen2)27, Sprague-Dawley (SD), and ASrAOGEN rat strains with respect to hemodynamic and baroreflex function. AOGEN: angiotensinogen, BRS: baroreflex sensitivity, RAS: renin-angiotensin system.

The renin-angiotensin system (RAS), and in particular angiotensin (Ang) II, is implicated in the impairment of arterial baroreflex function and reduction of heart rate variability (HRV) commonly associated with hypertension [1–4]. However, more recent studies suggest that a part of the deficit in sensitivity of the baroreflex function (BRS) in hypertension results from a reduction in Ang-(1–7), an alternative product of the RAS, rather than a frank increase in Ang II [5, 6]. Ang II blockade attenuates oxidant production and improves mitochondrial function in peripheral tissues in various experimental models of hypertension [7–10]. The contributions of Ang-(1–7) to the beneficial effects of Ang II blockers are increasingly recognized [11–16], but few studies have directly addressed the role of Ang-(1–7) in mitochondrial function. In this paper, we summarize (1) the role of Ang II in reactive oxygen species (ROS) generation and (2) the implication of ROS and redox-signaling on blood pressure, baroreflex, and mitochondrial function, with a particular focus on potential mechanisms for the counterbalancing role of Ang-(1–7) (Figure 1). Furthermore, we highlight the recent studies in transgenic rats with altered brain RAS (summarized in Figure 2) as a tool to study changes in brain ROS and signaling pathways in response to Ang peptides [Ang II and Ang-(1–7)] and their effect on BRS and mitochondrial function. The transgenic (mRen2)27 rat strain which overexpresses the murine Ren2 gene is hypertensive and has impaired BRS for control of heart rate (HR) with high levels of Ang II relative to Ang-(1–7) in the brain medullary tissue compared to the normotensive Sprague-Dawley (SD) rats [17, 18]. In contrast, transgenic rats with low glial angiotensinogen (ASrAOGEN) have lower mean arterial pressure (MAP) and HR suggesting decreased sympathetic nerve activity and enhanced BRS for control of HR relative to SD rats [19, 20]. While we would expect that both Ang II and Ang-(1–7) would be reduced in the glial cells, nonglial sources (neuronal and/or circulating) of angiotensinogen and Ang peptides appear to be intact in ASrAOGEN rats [21]. Tissue levels of Ang II relative to Ang-(1–7) in the medulla have not been reported; however, blockade of Ang II actions by an AT1 receptor antagonist revealed that there was no Ang II tone attenuating the BRS in anesthesized ASrAOGEN rats and we conclude that glia-derived Ang II is responsible for this action. In contrast, since blockade of endogenous Ang-(1–7) attenuates BRS in both younger and older anesthesized ASrAOGEN rats, a nonglial source of Ang-(1–7) likely contributes to the preservation of BRS in these animals [5, 22]. Thus, there appears to be low Ang II but maintenance of Ang-(1–7) tone contributing to the enhanced BRS seen in the medulla of these animals.

## 2. Angiotensin Peptides and ROS Generation in the Brain

Overactivation of the RAS in pathological conditions, such as hypertension, results in excessive ROS production through the prooxidant actions of Ang II [24, 25]. The contribution of cytoplasmic NADPH-oxidase-(NOX)-generated ROS by Ang II in neurogenic hypertension is well established [26–28]. Ang II also stimulates mitochondrial ROS; both as a result of cytoplasmic NOX-derived ROS or direct effects on mitochondria [29–32]. Scavenging mitochondrial ROS, through agents such as Mito-TEMPO that preferentially targets the mitochondria, prevents Ang II-induced hypertension in mice [31, 33]. Antioxidant therapies targeting mitochondria are suggested to disrupt the mitochondrial ROS-dependent stimulation of cytoplasmic NOX activity, thereby providing beneficial effects in hypertension [34]. However, recent studies show that the NOX isoforms are also present within mitochondria [35–37] and the contribution of mitochondrial NOX to overall mitochondrial ROS in hypertension is unknown. Molecular interventions to target specific NOX isoforms within the mitochondria or other cellular organelle are required to address this issue.

Ang-(1–7) has emerged as a major counter-regulatory peptide to Ang II actions and may serve to inhibit Ang II-stimulated ROS production through inhibiting NOX and/or increased ROS scavenging by augmenting antioxidant enzymes such as catalase [38, 39]. Indeed we find that higher Ang II actions relative to Ang-(1–7) in the brain dorsal medulla of hypertensive (mRen2)27 rats are associated with increased cytoplasmic NOX activity and ROS in isolated brain dorsal medullary mitochondria compared with the hypotensive ASrAOGEN [with higher Ang-(1–7) actions relative to Ang II] or the normotensive SD rats [40]. The levels of ROS were similar in the ASrAOGEN rats compared to SD rats under basal conditions suggesting that Ang-(1–7) may serve to inhibit NOX and/or activate antioxidant enzymes in response to Ang II stimulation. Although this concept is supported by several studies [38, 41], it has not been investigated directly in the brain to our knowledge. Thus, in this respect, it would be interesting to test whether blockade of endogenous Ang-(1–7) in ASrAOGEN rats will result in increased NOX activity/ROS levels in response to Ang II infusion/microinjection in the brain. 

## 3. ROS and Redox-Signaling in the Brain: Influences on Blood Pressure, Baroreflex, and Mitochondrial Function

Excessive ROS in brain contributes to increased sympathetic outflow [42, 43] and impairs mitochondrial [8, 31, 44, 45] and BRS function [46–48]. Our recent studies find that Ang-(1–7) via chronic ICV infusion improved vagal function independent of any blood pressure lowering effect in transgenic hypertensive (mRen2)27 rats [49]. This effect was in contrast to the response to AT_1_ receptor antagonist, candesartan, which normalized blood pressure but did not significantly improve the vagal indices of BRS or HRV. We have yet to determine the effect of these treatments on mitochondrial ROS, but neither treatment altered cytoplasmic NOX activity. Central infusion of the ROS scavenger tempol did not lower blood pressure or influence indices of baroreflex function, but significantly reduced cytoplasmic NOX activity, suggesting independence from ROS-related mechanisms for blood pressure lowering and autonomic nervous system balance in the hypertensive (mRen2)27 strain. However, we do not know whether tempol efficiently targets mitochondrial ROS or the extent that alterations in mitochondrial ROS would influence blood pressure and/or BRS in the transgenic rats. Dikalova and colleagues have reported blood pressure lowering effects of Mito-TEMPO in both Ang II-induced and DOCA salt hypertension in mice while a similar dose of tempol alone did not lower blood pressure in this study [33]. Furthermore, mitochondria are in close structural proximity to the endoplasmic reticulum (ER), and ER stress is implicated in mitochondrial dysfunction [50]. Indeed, the recent study by Young and colleagues link Ang II-induced hypertension to ER and oxidative stress in the brain [51]. These results provide a compelling case to investigate the effects of mitochondrial ROS, independent of cytoplasmic NOX.

**Figure 3:**
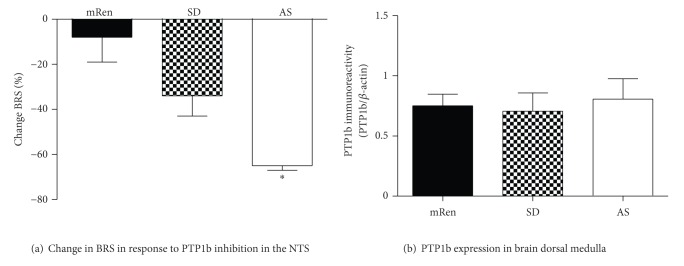
ASrAOGEN (AS) rats show significantly greater reduction in the evoked BRS for control of HR following inhibition of PTP1b (a), despite similar levels of the PTP1b protein in dorsal medulla of the three strains (b). Mean ± SEM (*n* = 4–6 per group for solitary tract nucleus (NTS) microinjections, *n* = 6 for Western blotting); *P < 0.05 versus (mRen2)27 rats. Data replotted from [23] for the Sprague-Dawley (SD) rats and original data presented for ASrAOGEN and (mRen2)27 [mRen] rats. Western blotting carried out as published for SD rats [23] and original data presented for ASrAOGEN and (mRen2)27 rats. Note the PTP1b antibody recognizes both phosphorylated and nonphosphorylated forms.

AngII/AT_1_ receptor/NOX-derived ROS are implicated in the activation of the MAP Kinases (MAPK) p38 and ERK1/2 that contribute to an impaired BRS and the pressor effects of Ang II in the RVLM [52–54]. A role for AT_1_ receptors and MAPKs in activation of mitochondrial apoptotic pathways in neural regulation of blood pressure and BRS is also apparent [54]. However, hypertensive (mRen2)27 rats which show an increased NOX activity in the brain dorsal medulla but not activated p38, ERK1/2, or JNK-1 in comparison to SD rats suggesting a lack of association of MAPK signaling pathways with high blood pressure or oxidative stress [40]. In contrast, (mRen2)27 rats have an upregulated phosphoinositol 3 kinase (PI3 K) pathway that contributes to the elevated MAP and impaired BRS [55]. Hypotensive ASrAOGEN rats with normal NOX activity exhibit reduced levels of phosphorylated ERK1/2 and JNK-1 but not p38 in the brain dorsal medulla [40]. These animals have significantly higher expression of MAPK phosphatase-1 [MKP-1, a negative regulator of MAPK signaling [40]] supporting the concept that Ang-(1–7) increases regulatory phosphatases that may buffer against acute Ang II-stimulated signaling. Indeed, ASrAOGEN rats show greater impairments in the BRS for control of HR following acute solitary tract nucleus inhibition of protein tyrosine phosphatase 1b (PTP1b), a negative regulator of the PI3 K pathway, suggestive of increased expression and/or activity of this phosphatase within the dorsal medulla (Figure 3(a)). However, protein expression of total PTP1b (phosphorylated and nonphosphorylated forms) is similar in the dorsal medulla among the three rat strains under baseline conditions (Figure 3(b)). Therefore, given the functional differences observed following inhibition of PTP1b activity, quantification of the phosphorylated active form of PTP1b is necessary to confirm increased PTP1b activity in the ASrAOGEN rats. Differences in ROS [higher in (mRen2)27 versus SD or AsrAOGEN] or the upstream-regulatory kinases/phosphatases can modulate the phosphatase activity by changes in phosphorylation status at a number of different sites, despite lack of changes in the total protein [56]. While an upregulation of phosphatase expression and activity within the dorsal medulla may contribute to the enhanced resting BRS in the ASrAOGEN animals relative to the normal baroreflex function in SD rats [23], the lack of endogenous PTP1b tone in transgenic (mRen2)27 rats (Figure 3(a)) could result in increased PI3 K activity that contributes to an impaired BRS and increased MAP in these animals [55]. 

An interesting paradox to the beneficial role of these regulatory phosphates is that both MKP-1 and PTP1b have negative effects on metabolic function [57, 58]. In this regard, global knockdown of these phosphatases improves insulin-sensitivity and prevents diet-induced obesity [59, 60]. MKP-1 is suggested to impair mitochondrial biogenesis in skeletal muscle in response to a high-fat diet through negative regulation of the p38 MAPKs [61]. However, ASrAOGEN rats that have increased activity of these phosphatases at least in dorsal medulla are resistant to diet-induced obesity and spared age-related decline in cardiovascular and metabolic functions [62, 63]. These animals have increased life-span and their phenotype mimics animals with long-term RAS blockade where improved mitochondrial function is reported [7, 62–64]. Whether the brain-specific actions of these phosphatases contribute to the beneficial metabolic effects in ASrAOGEN rats is of interest and currently unknown. Thus, further studies dissecting the role of these brain signaling pathways in regulating MAP, BRS, mitochondrial, and metabolic functions are warranted. 

**Figure 4:**
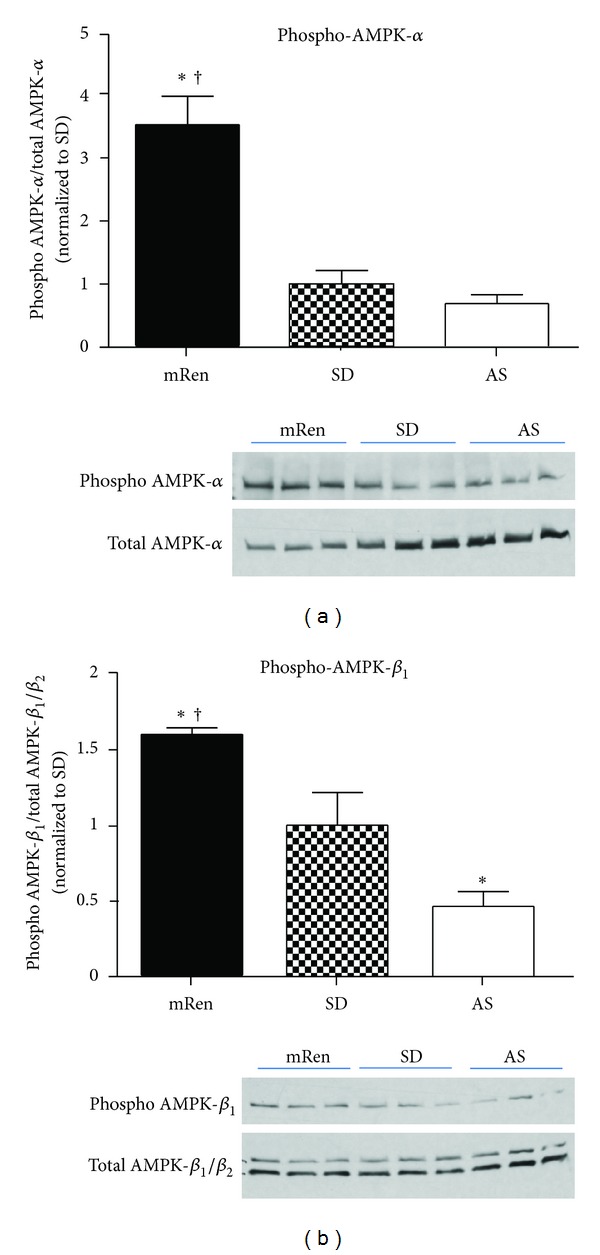
Hypertensive (mRen2)27 rats show significantly increased phosphorylated AMP-Kinase (AMPK) in the brain dorsal medulla. AMPK-*α* (a) and *β*_1_ (b) activities were measured by Western blot hybridization using phospho-specific antibodies (Cell Signaling) in brain dorsal medulla tissues from (mRen2)27 [mRen], Sprague-Dawley (SD), and ASrAOGEN (AS) rats. Top: Densitometry analyses of phoshorylated protein levels normalized to total AMPK-*α* and *β*_1_/*β*_2_; bottom: representative Western blots. Data are mean ± SEM (*n* = 3–6 per group); *P < 0.05 versus SD; ^†^ *P* < 0.05 versus AS rats.

**Figure 5:**
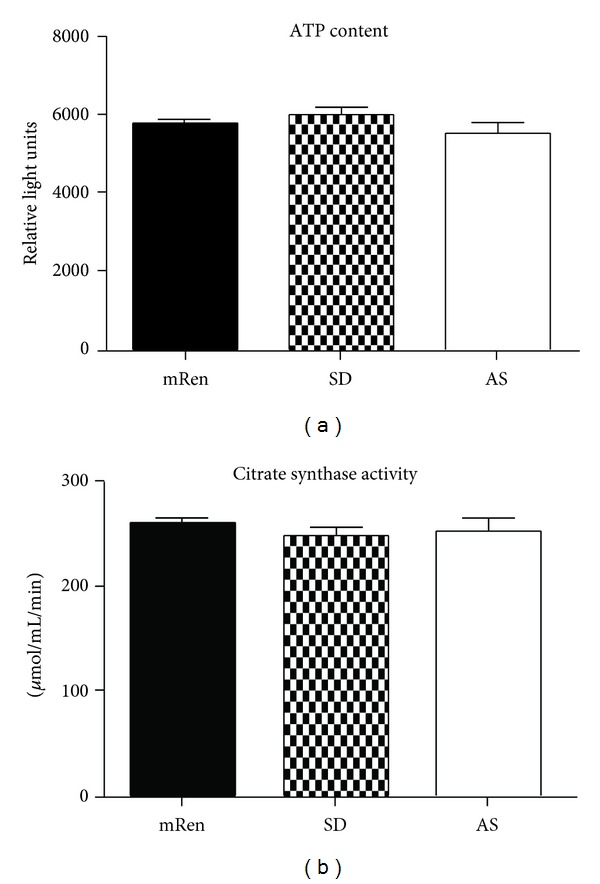
Brain dorsal medullary ATP levels (a) and mitochondrial content as measured using citrate synthase (b) was not different among hypertensive (mRen2)27 [mRen], Sprague-Dawley (SD) or hypotensive ASrAOGEN (AS) rats. ATP levels were determined using a chemiluminescent assay (Promega) and citrate synthase activity was measured using the assay kit (Sigma) in tissue homogenates. Mean ± SEM (*n* = 6 per group).

Altered mitochondrial oxidant and/or energy levels are associated with a stimulated AMP-activated protein kinase (AMPK) pathway that is activated in response to depleted cellular energy levels, to restore mitochondrial biogenesis and ATP levels [65, 66]. AMPK was significantly activated (phosphorylated AMPK-*α* and *β*_1_ subunits, Figures 4(a) and 4(b), resp.,) in the dorsal medulla of transgenic (mRen2)27 rats that exhibit increased cytoplasmic NOX activity and ROS levels in the brain dorsal medullary mitochondria relative to SD rats [40]. While we expected lower ATP levels (Figure 5(a)) and the mitochondrial content/number (assessed indirectly using the marker of mitochondrial health or activity, citrate synthase enzyme activity, Figure 5(b)) in the (mRen2)27 rats, these markers were not different in the dorsal medulla of the three strains. Therefore, activation of AMPK in the (mRen2)27 rats may represent a compensatory response to restore normal ATP and mitochondrial activity in the hypertensive strain in the face of increased ROS. Additional studies are necessary to address whether (1) blockade of AMPK activation lowers mitochondrial content and depletes ATP levels and (2) targeting mitochondrial ROS improves MAP, BRS, and mitochondrial function in the hypertensive (mRen2)27 rats. 

## 4. Conclusions and Perspectives

Mitochondria-derived ROS which often accompanies impaired autonomic function is an emerging therapeutic target in hypertension [31, 33, 34, 45–47]. Increased cellular ROS may manifest as impaired BRS for the control of HR and reduced HRV (decreased parasympathetic outflow or vagal tone); and these indices of autonomic imbalance are associated with increased overall mortality, independent of blood pressure. Therefore, determining the key cellular mechanisms underlying the beneficial actions of Ang-(1–7) (such as altered kinase-phosphatase signaling) in influencing baroreflex function may help elucidate new therapeutic targets for reducing cardiometabolic pathologies. While Ang-(1–7) has been investigated for its role in attenuation of ROS, studies specifically addressing the mitochondria are lacking and few investigators are studying the interactions in brain. Thus, targeting improved vagal and mitochondrial function in addition to MAP may provide better target organ protection than lowering blood pressure alone, leading to reductions in all cause mortality.
